# Supplementary material for: Incomplete antiviral treatment may induce longer durations of viral shedding during SARS-CoV-2 infection
Source: Life Sci Alliance. 2021 Aug 3;4(10):e202101049. doi: 10.26508/lsa.202101049 (PMC8340032; doi:10.26508/lsa.202101049)
Supplement: Supplementary file 1 [file LSA-2021-01049_TableS1.docx]

**Table S1. Individual estimated parameters and initial values in nose and throat swabs**

| **Monkey ID** | $\boldsymbol{r}$ | $\boldsymbol{\beta}$ | $\boldsymbol{\delta}$ | $\boldsymbol{\varepsilon}$ | $\boldsymbol{V(0)}$ |
| --- | --- | --- | --- | --- | --- |
| Without treatment | | | | | |
| RM A | $18.2$ | $1.73\times{10}^{-6}$ | $0.964$ | $-$ | $2.71\times{10}^{3}$ |
| RM B | $18.3$ | $1.46\times{10}^{-6}$ | $0.968$ | $-$ | $1.58\times{10}^{3}$ |
| RM C | $18.1$ | $1.74\times{10}^{-6}$ | $0.912$ | $-$ | $2.70\times{10}^{3}$ |
| RM D | $18.1$ | $2.09\times{10}^{-6}$ | $1.18$ | $-$ | $4.43\times{10}^{3}$ |
| RM 7 | $18.1$ | $2.24\times{10}^{-6}$ | $1.49$ | $-$ | $7.09\times{10}^{3}$ |
| RM 8 | $18.1$ | $2.21\times{10}^{-6}$ | $1.45$ | $-$ | $3.89\times{10}^{3}$ |
| RM 9 | $18.2$ | $1.92\times{10}^{-6}$ | $1.15$ | $-$ | $3.75\times{10}^{3}$ |
| RM 10 | $18.3$ | $1.47\times{10}^{-6}$ | $1.08$ | $-$ | $1.38\times{10}^{3}$ |
| RM 11 | $18.2$ | $1.75\times{10}^{-6}$ | $1.18$ | $-$ | $2.46\times{10}^{3}$ |
| RM 12 | $18.2$ | $1.49\times{10}^{-6}$ | $1.14$ | $-$ | $1.52\times{10}^{3}$ |
| RM A | $2.94$ | $6.39\times{10}^{-6}$ | $1.15$ | $-$ | $7.33\times{10}^{3}$ |
| RM D | $2.82$ | $6.65\times{10}^{-6}$ | $1.19$ | $-$ | $5.43\times{10}^{2}$ |
| RM 7 | $2.84$ | $6.79\times{10}^{-6}$ | $1.18$ | $-$ | $8.16\times{10}^{2}$ |
| RM 8 | $2.8$ | $7.25\times{10}^{-6}$ | $1.23$ | $-$ | $50.8$ |
| RM 9 | $2.98$ | $8.26\times{10}^{-6}$ | $1.16$ | $-$ | $2.31\times{10}^{2}$ |
| RM 10 | $2.93$ | $5.93\times{10}^{-6}$ | $1.05$ | $-$ | $1.54\times{10}^{4}$ |
| RM 11 | $2.87$ | $6.78\times{10}^{-6}$ | $1.14$ | $-$ | $9.07\times{10}^{3}$ |
| RM 12 | $2.9$ | $6.64\times{10}^{-6}$ | $1.06$ | $-$ | $8.46\times{10}^{5}$ |
| With treatment | | | | | |
| RM 1 | $18.5$ | $1.57\times{10}^{-6}$ | $1.08$ | $0.614$ | $2.71\times{10}^{3}$ |
| RM 2 | $16.9$ | $1.65\times{10}^{-6}$ | $1.36$ | $0.887$ | $1.58\times{10}^{3}$ |
| RM 3 | $18.7$ | $1.26\times{10}^{-6}$ | $0.996$ | $0.515$ | $2.70\times{10}^{3}$ |
| RM 4 | $18.4$ | $1.57\times{10}^{-6}$ | $1.2$ | $0.572$ | $4.43\times{10}^{3}$ |
| RM 5 | $18.5$ | $1.53\times{10}^{-6}$ | $1.07$ | $0.579$ | $7.09\times{10}^{3}$ |
| RM 6 | $18.8$ | $1.41\times{10}^{-6}$ | $1.06$ | $0.540$ | $3.89\times{10}^{3}$ |
| RM 1 | $2.90$ | $6.70\times{10}^{-6}$ | $1.31$ | $0.576$ | $3.75\times{10}^{3}$ |
| RM 2 | $2.90$ | $6.66\times{10}^{-6}$ | $1.3$ | $0.488$ | $1.38\times{10}^{3}$ |
| RM 3 | $2.91$ | $6.57\times{10}^{-6}$ | $1.29$ | $0.515$ | $2.46\times{10}^{3}$ |
| RM 4 | $2.90$ | $6.60\times{10}^{-6}$ | $1.3$ | $0.564$ | $2.71\times{10}^{3}$ |
| RM 5 | $2.91$ | $6.61\times{10}^{-6}$ | $1.29$ | $0.518$ | $1.58\times{10}^{3}$ |
| RM 6 | $2.87$ | $6.71\times{10}^{-6}$ | $1.55$ | $0.865$ | $2.70\times{10}^{3}$ |
