# Supplementary material for: Incomplete antiviral treatment may induce longer durations of viral shedding during SARS-CoV-2 infection
Source: Life Sci Alliance. 2021 Aug 3;4(10):e202101049. doi: 10.26508/lsa.202101049 (PMC8340032; doi:10.26508/lsa.202101049)
Supplement: Supplementary file 5 [file LSA-2021-01049_TableS2.docx]

**Table S2. Estimated parameters (fixed effect) for SARS-CoV-2 infection in lung**

| Parameter Name | Symbol (Unit) | BAL |
| --- | --- | --- |
| Parameters in the model | | |
| Maximum rate constant for viral replication | $\gamma$ (day^-1^) | $47.3$^#^ |
| Rate constant for virus infection | $\beta$ $(($copies/ml)^-1^ day^-1^) | ${3.51\times10}^{-7}$^#^ |
| Death rate of infected cells | $\delta$ (day^-1^) | $1.14$ |
| Efficacy of blocking virus production by RDV | $\varepsilon$ | $0.618$ |
| Viral load at virus inoculation | $V(0)$ (copies/ml) | ${2.86\times10}^{3}$ |
| Quantities derived from the parameters | | |
| Within-host basic reproduction number | $R_{0}$ ($=\gamma/\delta$) | $41.5$^#^ |
| Malthusian parameter | $M$ ($=\gamma-\delta$) | $46.2$^#^ |

^#^ Statistically different from nose (the Wald test).
