# Supplementary material for: Incomplete antiviral treatment may induce longer durations of viral shedding during SARS-CoV-2 infection
Source: Life Sci Alliance. 2021 Aug 3;4(10):e202101049. doi: 10.26508/lsa.202101049 (PMC8340032; doi:10.26508/lsa.202101049)
Supplement: Supplementary file 6 [file LSA-2021-01049_TableS3.docx]

**Table S3. Individual estimated parameters and initial values in lung**

| **Monkey ID** | $\boldsymbol{r}$ | $\boldsymbol{\beta}$ | $\boldsymbol{\delta}$ | $\boldsymbol{\varepsilon}$ | $\boldsymbol{V(0)}$ |
| --- | --- | --- | --- | --- | --- |
| Without treatment | | | | | |
| RM A | $47.4$ | $3.35\times{10}^{-7}$ | $1.14$ | $-$ | $2.60\times{10}^{3}$ |
| RM B | $47.1$ | $3.99\times{10}^{-7}$ | $1.25$ | $-$ | $3.70\times{10}^{3}$ |
| RM C | $47.4$ | $3.37\times{10}^{-7}$ | $1.13$ | $-$ | $2.64\times{10}^{3}$ |
| RM D | $47.3$ | $3.36\times{10}^{-7}$ | $1.12$ | $-$ | $2.75\times{10}^{3}$ |
| RM 7 | $47.4$ | $3.24\times{10}^{-7}$ | $1.10$ | $-$ | $2.67\times{10}^{3}$ |
| RM 8 | $47.0$ | $3.95\times{10}^{-7}$ | $1.25$ | $-$ | $3.85\times{10}^{3}$ |
| RM 9 | $47.1$ | $3.57\times{10}^{-7}$ | $1.25$ | $-$ | $2.70\times{10}^{3}$ |
| RM 10 | $47.5$ | $3.32\times{10}^{-7}$ | $1.12$ | $-$ | $2.65\times{10}^{3}$ |
| RM 11 | $47.4$ | $3.48\times{10}^{-7}$ | $1.10$ | $-$ | $2.96\times{10}^{3}$ |
| RM 12 | $47.2$ | $3.56\times{10}^{-7}$ | $1.23$ | $-$ | $2.62\times{10}^{3}$ |
| With treatment | | | | | |
| RM 1 | $47.4$ | $3.43\times{10}^{-7}$ | $1.26$ | $0.623$ | $2.66\times{10}^{3}$ |
| RM 2 | $47.3$ | $3.66\times{10}^{-7}$ | $1.36$ | $0.624$ | $3.13\times{10}^{3}$ |
| RM 3 | $46.9$ | $4.33\times{10}^{-7}$ | $1.65$ | $0.618$ | $4.53\times{10}^{3}$ |
| RM 4 | $47.3$ | $3.54\times{10}^{-7}$ | $1.41$ | $0.611$ | $2.39\times{10}^{3}$ |
| RM 6 | $47.0$ | $4.27\times{10}^{-7}$ | $1.48$ | $0.617$ | $4.60\times{10}^{3}$ |
