# Supplementary material for: Incomplete antiviral treatment may induce longer durations of viral shedding during SARS-CoV-2 infection
Source: Life Sci Alliance. 2021 Aug 3;4(10):e202101049. doi: 10.26508/lsa.202101049 (PMC8340032; doi:10.26508/lsa.202101049)
Supplement: Supplementary file 9 [file LSA-2021-01049_TableS4.docx]

**Table S4. Estimated parameters (fixed effect) for SARS-CoV-2 infection in nose and throat by fitting the viral dynamics with eclipse phase**

| Parameter Name | Symbol (Unit) | Nose | Throat | BAL |
| --- | --- | --- | --- | --- |
| Maximum rate constant for viral replication | $\gamma$ (day^-1^) | $1.96\times{10}^{7}$ | $1.67\times{10}^{6}$ | $2.06\times{10}^{8}$ |
| Rate constant for virus infection | $\beta$ $(($copies/ml)^-1^ day^-1^) | ${2.3\times10}^{-5}$ | | |
| Death rate of infected cells | $\delta$ (day^-1^) | $1.09$ | | |
| Efficacy of blocking virus production by RDV | $\varepsilon$ | $0.889$ | | |
| Transition rate from eclipse phase^#^ | $k$ (day^-1^) | $3.00$ | | |
| Viral load at virus inoculation | $V(0)$ (copies/ml) | ${4.20\times10}^{2}$ | | |

^#^This parameter is fixed according to (53)
