# Supplementary material for: Incomplete antiviral treatment may induce longer durations of viral shedding during SARS-CoV-2 infection
Source: Life Sci Alliance. 2021 Aug 3;4(10):e202101049. doi: 10.26508/lsa.202101049 (PMC8340032; doi:10.26508/lsa.202101049)
Supplement: Supplementary file 11 [file LSA-2021-01049_TableS5.docx]

**Table S5. Estimated parameters (fixed effect) for SARS-CoV-2 infection in nose and throat by fitting the viral dynamics with multiple target cell**

| Parameter Name | Symbol (Unit) | Nose | Throat | BAL |
| --- | --- | --- | --- | --- |
| Maximum rate constant for viral replication for first target cell | $\gamma_{1}$ (day^-1^) | $18.9$ | $4.17$ | $22.1$ |
| Maximum rate constant for viral replication for second target cell | $\gamma_{2}$ (day^-1^) | $0.128$ | | |
| Rate constant for virus infection for first target cell | $\beta_{1}$ $(($copies/ml)^-1^ day^-1^) | ${1.22\times10}^{-6}$ | ${9.67\times10}^{-6}$ | ${1.07\times10}^{-7}$ |
| Rate constant for virus infection for second target cell | $\beta_{2}$ $(($copies/ml)^-1^ day^-1^) | $2.81{\times10}^{-6}$ | | |
| Death rate of infected cells | $\delta$ (day^-1^) | $1.26$ | | |
| Efficacy of blocking virus production by RDV | $\varepsilon$ | $0.584$ | | |
| Viral load at virus inoculation | $V(0)$ (copies/ml) | ${2.06\times10}^{3}$ | | |
| Relative fraction of first uninfected target cell population at virus inoculation | $f_{1}\left( 0 \right)$ | $0.613$ | | |
| Relative fraction of second uninfected target cell population at virus inoculation | $f_{2}\left( 0 \right)$ | $0.387$ | | |
