# Supplementary material for: Incomplete antiviral treatment may induce longer durations of viral shedding during SARS-CoV-2 infection
Source: Life Sci Alliance. 2021 Aug 3;4(10):e202101049. doi: 10.26508/lsa.202101049 (PMC8340032; doi:10.26508/lsa.202101049)
Supplement: Supplementary file 13 [file LSA-2021-01049_TableS6.docx]

**Table S6. Estimated parameters (fixed effect) for SARS-CoV-2 infection in nose and throat by fitting the viral dynamics with innate immune response**

| Parameter Name | Symbol (Unit) | Nose | Throat | BAL |
| --- | --- | --- | --- | --- |
| Maximum rate constant for viral replication | $\gamma$ (day^-1^) | $38$ | $12$ | $2000$ |
| Rate constant for virus infection | $\beta$ $(($copies/ml)^-1^ day^-1^) | ${1.68\times10}^{-6}$ | ${2.23\times10}^{-6}$ | ${1.02\times10}^{-6}$ |
| Death rate of infected cells | $\delta$ (day^-1^) | $1.15$ | | |
| Efficacy of blocking virus production by RDV | $\varepsilon$ | $0.865$ | | |
